# Functional Rescue of a Kidney Anion Exchanger 1 Trafficking Mutant in Renal Epithelial Cells

**DOI:** 10.1371/journal.pone.0057062

**Published:** 2013-02-27

**Authors:** Carmen Y. S. Chu, Jennifer C. King, Mattia Berrini, R. Todd Alexander, Emmanuelle Cordat

**Affiliations:** Department of Physiology, University of Alberta, Edmonton, Alberta, Canada; Tohoku University, Japan

## Abstract

Mutations in the *SLC4A1* gene encoding the anion exchanger 1 (AE1) can cause distal renal tubular acidosis (dRTA), a disease often due to mis-trafficking of the mutant protein. In this study, we investigated whether trafficking of a Golgi-retained dRTA mutant, G701D kAE1, or two dRTA mutants retained in the endoplasmic reticulum, C479W and R589H kAE1, could be functionally rescued to the plasma membrane of Madin-Darby Canine Kidney (MDCK) cells. Treatments with DMSO, glycerol, the corrector VX-809, or low temperature incubations restored the basolateral trafficking of G701D kAE1 mutant. These treatments had no significant rescuing effect on trafficking of the mis-folded C479W or R589H kAE1 mutants. DMSO was the only treatment that partially restored G701D kAE1 function in the plasma membrane of MDCK cells. Our experiments show that trafficking of intracellularly retained dRTA kAE1 mutants can be partially restored, and that one chemical treatment rescued both trafficking and function of a dRTA mutant. These studies provide an opportunity to develop alternative therapeutic solutions for dRTA patients.

## Introduction

Distal renal tubular acidosis (dRTA) is a dominantly or recessively inherited disease characterized by development of metabolic acidosis, inability to acidify urine, hypokalemia, nephrocalcinosis, and eventually renal failure if untreated [1]. Mutations in the *SLC4A1* gene encoding anion exchanger 1 (AE1) can cause dRTA, often due to altered trafficking of the mutant protein [2]. AE1 is expressed at the basolateral membrane of type A intercalated cells (kAE1) in the renal collecting duct, where it mediates electroneutral chloride/bicarbonate exchange and thus participates in the fine-tuning of acid-base homeostasis. AE1 is also expressed in red blood cells (eAE1) where it plays a crucial role in respiration. Up to 16 mutations in the *SLC4A1* gene have been identified that cause dRTA [3]–[6]. Interestingly, some mutations in the *SLC4A1* gene cause hereditary spherocytosis (HS), a common form of inherited haemolytic anemia, but the same mutation rarely causes both HS and dRTA. It is possible that cell-specific chaperones expressed either in red blood cells, such as glycophorin A (GPA) [7], or kidney cells promote proper folding of dRTA or HS mutants, respectively. The restricted expression of these chaperones would then explain the lack of dRTA symptoms in HS patients and vice-versa. Alternatively, stringency of the quality-control machineries may differ between kidneys and red blood cells, resulting in differential surface expression of AE1 mutants depending on the cell type. Recent advances showed that dominant dRTA mutants retain wild-type (WT) kAE1 intracellularly, while recessive dRTA mutants have their trafficking rescued by co-expression with WT kAE1 [8].

kAE1 is a dimeric membrane protein with cytoplasmic amino- and carboxyl-termini, that bind to various cytosolic proteins including carbonic anhydrase II, glyceraldehyde-3 phopshate dehydrogenase (GAPDH), integrin-linked kinase, nephrin and subunits of adaptor protein complexes [9]–[13]. The transmembrane domain carries the anion exchange activity and a single N-glycosylation site in the fourth extracellular loop. The transmembrane domain also contains the binding site for stilbene inhibitor derivatives such as 4,4′-Diisothiocyano-2,2′-dihydrostilbenedisulfonate (H_2_DIDS) and 4-acetamido-4′-isothiocyanostilbene-2,2′-disulfonate (SITS) [14].

Newly synthesized membrane proteins are processed through the endoplasmic reticulum (ER) where complex quality-control machinery ensures proper protein folding prior to release and further trafficking to the Golgi, trans-Golgi network (TGN) and the plasma membrane. Many mutated membrane proteins retain partial function but do not traffic to their final destination, and are prematurely degraded by the ER- and post-ER quality-control machinery [15], [16]. Seven out of the eight eAE1 mutants isolated from dRTA patients’ red blood cells displayed greater than 50% of WT sulfate influx [2], [17]–[20], supporting that the major hindrance of these mutants is mis-trafficking rather than mis-folding [21]. Furthermore, these mutants are often functional when expressed in *Xenopus* oocytes [2], but they are consistently retained intracellularly in renal epithelial cells.

Rescuing mis-trafficking has been attempted on many mutant membrane proteins over the recent years. One striking example is the rescue of the most common mutant protein leading to Cystic Fibrosis, the deltaF508 cystic fibrosis transmembrane conductance regulator (CFTR) by the corrector VX-809 [22]. This chemical chaperone appears to act by specifically improving the folding of a portion of the deltaF508 CFTR mutant in the ER, allowing the protein to escape the ER quality-control machinery and traffic to the plasma membrane of human bronchial epithelial cells where it is functional. Earlier studies described the successful rescue of mis-trafficking mutant proteins by incubating living cells in conditions known to stabilize proteins in their native environment such as low temperature (26–30°C) [23] or chemical chaperones [24]. These chemical or low temperature incubations have become standard rescuing methods for initial studies on restoration of mis-trafficking mutant proteins. A recent study reported the cell surface rescue of two dominant R589H and R901X kAE1 dRTA mutants after disrupting chaperones interaction in Madin-Darby Canine Kidney (MDCK) cells [25]. In contrast, Toye and colleagues were unsuccessful in their attempt to increase cell surface trafficking of kAE1 mutants, including the ER retained R589H and S613F dRTA mutants using sodium butyrate or low temperature incubations in MDCK I cells [26]. However, none of these studies addressed whether rescued mutants are functional at the cell surface. The goal of our study was to determine whether basolateral trafficking and function of a Golgi retained, recessive dRTA mutant – G701D kAE1, an ER-retained, dominant dRTA mutant – R589H kAE1 and an ER-retained, recessive dRTA mutant – C479W kAE1 could be rescued to the plasma membrane of renal epithelial cells using a variety of chemical or low temperature treatments. The G701D recessive dRTA mutant is most commonly found in Southeast Asia, either in the homozygous state or in the compound heterozygous state with S773P, A858D, E522K, C479W or the Southeast Asian Ovalocytosis mutant [4], [20], [27]–[29]. The G701D kAE1 mutant is retained in the Golgi of renal epithelial cells [8]. When co-expressed with GPA in *Xenopus* oocytes, this mutant was functionally detected at the cell surface [30]. Similar to G701D, when expressed in Human Embryonic Kidney (HEK) 293 cells, the dominant R589H kAE1 and R589H eAE1 mutants were both retained intracellularly despite folding similar to kAE1 WT [31]. In *Xenopus* oocytes, although both isoforms were approximately 50% functional compared to kAE1 WT, their functional activity improved when co-expressed with GPA [2]. On the other hand, the recently identified C479W kAE1 mutant was found to be inactive in *Xenopus* oocytes and retained in the ER of renal epithelial cells [27]. This C479W kAE1 mutant was chosen as a non-functional negative control. Therefore, in our current study, we treated MDCK cells expressing WT, G701D, R589H or C479W kAE1 dRTA mutants with chemical (glycerol, DMSO) and pharmacological chaperones (VX-809) or incubated them at low temperature (30°C) to determine whether these treatments rescued the surface localization and functional activity of mutant kAE1 at the basolateral plasma membrane.

## Materials and Methods

### AE1 Mutant Constructs

cDNAs encoding WT, G701D, C479W, and R589H kAE1 were cloned into the viral vector pFB-Neo (Stratagene), as described previously [8], [27]. These cDNAs all contain the sequence encoding a haemagglutinin (HA) epitope in the third extracellular loop at position 557, previously shown to have no effect on normal folding and function of kAE1 [8], [32].

### Transfections and Viral Infections

MDCK (CCL-34) and human embryonic kidney 293 (CRL-11268) cells were purchased from the American Type Culture Collection (ATCC, Manassas, USA). Viral infections were performed as previously described [8]. Briefly, human embryonic kidney 293 cells were transiently transfected with p-VPack-GP, p-VPack-VSV-G and pFB neo kAEI-HA557 WT or mutant plasmids (Stratagene) using FuGENE6™ (Roche Applied Science). The supernatant containing the virus was collected and added to sub-confluent MDCK cells with 8 µg/ml hexadimethrine bromide (polybrene; Sigma). Twenty-four hours after infection, cells were selected with 1 mg/ml of geneticin. Expression levels of kAE1 in infected MDCK cells decreases over a two to three week period (on average, 6 passages), therefore infected cells were not used for periods longer than three weeks.

### Treatments

For rescue treatments, non-confluent or polarized MDCK cells expressing the WT or mutant kAE1 proteins were treated with the indicated concentrations of glycerol, DMSO or incubated at 30°C for either 4 or 16 hours prior to lysate preparation or immunostaining. Twenty-four hour incubations with 3 µM of VX-809 (Selleck) at 37°C were also used to treat cells expressing mutant kAE1. As DMSO was the vehicle for VX-809, treatments with 3 µM VX-809 introduced a final 0.4% DMSO, a concentration that did not significantly improve trafficking of kAE1 WT or G701D mutant. All treatments were applied in medium DMEM F12 containing 0.05 mg/ml penicillin/streptomycin, 10% FBS and 1 mg/ml geneticin (Invitrogen). To block protein synthesis, MDCK cells expressing WT or mutant kAE1 proteins were treated with 10 µg/ml cycloheximide (CHX; Sigma) in DMEM F12 containing 0.05 mg/ml penicillin/streptomycin, 10% FBS and 1 mg/ml geneticin. CHX treatment allowed us to study a specific pool of proteins inside the cell under various conditions. The integrity of tight junctions in control conditions or after a 16 hours treatment was verified by measuring trans-epithelial electrical resistances (TEER) four days after plating confluent MDCK cells on semi-permeable filters. TEER values were: 588, 625, 646 and 760 ohms.cm^2^ in control, DMSO, glycerol and 30°C treatments, respectively. Additionally, immunolabelling results confirmed the normal localization of the tight junction protein *Zona occludens*-1 in control and treated conditions (data not shown).

### Lysate Preparation and Immunoblots

MDCK cells expressing WT or mutant kAE1 were lysed with PBS (140 mM NaCl, 2.7 mM KCl, 10 mM Na_2_HPO_4_, and 1.8 mM KH_2_PO_4_ at pH 7.4) containing 1% Triton X-100 and protease inhibitors leupeptin (Sigma), aprotinin (Sigma), pepstatin (Sigma), and PMSF (Sigma). Protein concentrations in cell lysates were quantified using the BCA assay (using bovine serum albumin (BSA); Sigma). Five to fifteen µg (amount varies between experiments) of proteins were diluted in 2X Laemmli sample buffer (Bio-Rad) and loaded onto 8% SDS-PAGE gels. Proteins were then transferred to nitrocellulose or PVDF membranes (Bio-rad). The membranes were blocked with 3% milk in TBST (5 mM Tris base, 15 mM NaCl, 0.1% Tween-20), incubated in 1% skim milk in TBST containing mouse anti-HA (Covance) primary antibody, followed by anti-mouse secondary antibody coupled to horseradish peroxidase (HRP). Probed proteins were detected with enhanced BM chemiluminescence Blotting Substrate (POD; Roche) or ECL Plus/Prime Western Blotting Detection System (Amersham, GE) on film (Kodak). Intensities of the bands were examined using ImageJ software. For measuring the percentage of kAE1 carrying complex oligosaccharide in either untreated or treated cells, the intensity of bands corresponding to kAE1 carrying complex oligosaccharides (upper band, measured using ImageJ) was divided by the one calculated from both kAE1 carrying high mannose and complex oligosaccharides (bottom and upper band).

### Immunocytochemistry

Confluent MDCK cells expressing WT or mutant kAE1 were grown on semi-permeable Transwell polycarbonate filters (Corning) to confluency for four to five days, fixed with 4% paraformaldehyde (Canemco Supplies) in PBS followed by incubation with 100 mM glycine in PBS (pH 8.5) to quench non-specific fluorescence. The kAE1 proteins located at the cell surface were detected with mouse anti-HA primary antibody (Covance) followed by an anti-mouse antibody coupled to Cy3 (Jackson Immunoresearch Laboratories). All the antibodies were added simultaneously from the apical and basolateral side of the cell monolayer. Cells were then permeabilized with 0.2% Triton X-100 in PBS for 10 minutes and incubated with mouse anti-HA primary antibody again followed by a secondary Alexa 488 antibody (Invitrogen Molecular Probes) to detect intracellular kAE1. 4′-6-Diamidino-2-phenylindole (DAPI, Sigma) was used to stain the nucleus. Samples were visualized with WaveFx Spinning Disk confocal microscope using the 60×oil immersion objective. For quantification of kAE1cell surface expression, we randomly counted the number of cells that displayed red (cell surface) staining out of cells displaying green staining (total expression) from three independent experiments.

### Functional Assay

MDCK cells expressing WT or mutant kAE1 were grown on 11×7.5 mm glass coverslips in 6 cm^2^ dishes until 70% confluent. Cells were treated with 1% DMSO or 1% glycerol at 37°C or at 30°C for 16 hours and VX-809 for 24 hours. The complete procedure has been previously described [33]. Briefly, the coverslips were rinsed three times with serum-free OptiMEM medium (Gibco), followed by incubation with 10 µM BCECF-AM (Sigma) for 10 minutes at 37°C or 30°C for the temperature treatment. Coverslips were then placed in fluorescence cuvettes and the cells were perfused with: Ringer’s buffer (5 mM glucose, 5 mM potassium gluconate, 1 mM calcium gluconate, 1 mM magnesium sulphate, 10 mM Hepes, 2.5 mM NaH_2_PO_4_, 25 mM NaHCO_3_) containing 140 mM chloride, followed by a chloride free medium containing 140 mM gluconate to induce intracellular alkalization. The experiments were performed at room temperature. BCECF intracellular fluorescence was calibrated with buffers at pH 6.5, 7.5, or 7.0 containing 100 µM nigericin sodium salt (Sigma). The Ringer’s buffers were continuously perfused with air: CO_2_ (19∶ 1). A Photon Technologies International (PTI) (London, Ontario, Canada) fluorimeter was used to read the fluorescence fluctuations produced from the samples. Excitation wavelengths 440 and 490 nm and emission wavelength 510 nm were used (calibrated to the fluorimeter). Transport rates of the cells were determined by linear regression of the initial H^+^ flux (first 30 seconds), normalized to pH calibration measurements. All measurements were done using Felix software. The intrinsic buffer capacities of the cells were measured according to [34] and were found to equal 11.8±3.0 (n = 8, ± SEM), 20.4±2.9 (n = 6, ± SEM) and 21.2±4.0 (n = 7, ± SEM) mM/pH for cells treated with DMSO, glycerol or incubated at 30°C, respectively, compared to 16.8±6.8 (n = 6, ± SEM) in control conditions. The difference between intrinsic buffer capacities did not reach statistical significance between control and treated cells.

### SITS Affi-gel Binding Assay

MDCK cells expressing WT or mutant kAE1 were lysed in PBS containing 1% Triton X-100 and protease inhibitors leupeptin (Sigma), aprotinin (Sigma), pepstatin (Sigma), and PMSF (Sigma). Protein concentrations in cell lysates were quantified using the BCA assay (using bovine serum albumin (BSA); Sigma) and volumes of cell lysates were adjusted to a final 1 mg/ml concentration. 4-acetamido-4′-isothiocyanostilbene-2,2′-disulfonate (SITS)-Affi-gel was prepared according to [35]. Cell lysates (100 µl) were combined to 25 µl of SITS-Affi-gel in 0.1% Triton X-100, 228 mM sodium citrate buffer pH 7.1, with and without 1 mM of free anion transport inhibitor H_2_DIDS. The mixture was incubated for 1 hour at 4°C. After collecting the resin by centrifugation and three washes with 0.1% Triton X-100, 228 mM citrate buffer pH 7.1, proteins were eluted by addition of 75 µl of 2×Laemmli sample buffer (Bio-Rad). Total and bound fractions were analyzed by immunoblotting, using a mouse anti-HA antibody and an anti-mouse antibody coupled to HRP.

### Statistical Analysis

Experimental results are summarized as mean ± SEM. All statistical comparisons were made using unpaired Student’s *t* test, unless stated otherwise. A *P* value less than 0.05 was considered statistically significant.

## Results

### Trafficking of G701D and R589H Intracellular Mutants can be Rescued by Chemical or Low Temperature Incubations

In this study, we examined whether the trafficking of one dominant (R589H) and two recessive (C479W and G701D) dRTA mutants of kAE1 could be restored to the plasma membrane by treating cells with DMSO, glycerol, low temperature incubations or the pharmacological chaperone VX-809.

To this end, we studied the trafficking of WT and mutant kAE1 carrying an extracellular hemaglutinin epitope when expressed in MDCK cells. By quantifying the amount of kAE1 detectable after incubation with the protein synthesis inhibitor CHX, we determined the half-lives of WT, G701D, R589H and C479W kAE1 to be 23 hours, 4 hours, 1 hour and 5 hours respectively, in MDCK cells (data not shown). These numbers are in agreement with the previously published half-lives measured for the same mutants in HEK and MDCK cells [25], [31]. Thus, treatment for 16 hours should be sufficient to show detectable effects on each of the mutant tested.

**Figure 1:**
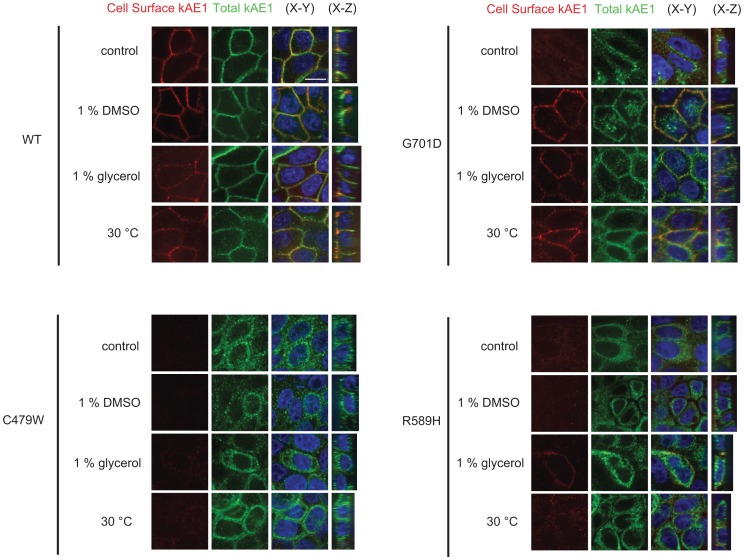
Low temperature incubations and chemical chaperones can partially rescue trafficking of dRTA mutants. MDCK cells expressing kAE1 WT or mutants were grown on semi-permeable filters until polarized, then treated either in control conditions, with 1% DMSO, 1% glycerol or at 30°C for 16 hours, fixed, blocked and stained with a mouse anti-HA antibody followed by an anti-mouse antibody coupled to Cy3 (red) fluorophore. Cells were then permeabilized, blocked and incubated with mouse anti-HA antibody again followed by an anti-mouse antibody coupled to Alexa 488 fluorophore (green). Yellow staining corresponds to overlap between green and red staining. The size bar represents 10 µm. The samples were examined with a spinning disk microscope using a 60×oil immersion objective. The images displayed are representative of 3 independent experiments.

Subsequent to treatment, WT and mutant kAE1 proteins expressed in polarized cells were examined by immunofluorescence. The HA epitope is located in the third extracellular loop for detection [32], consequently, it allowed us to distinguish between basolateral and intracellular kAE1 by performing a two-step immunostaining prior to and after permeabilization (see Methods for details) (Fig. 1). Red staining corresponds to kAE1 present at the plasma membrane while green staining shows total surface kAE1 after permeabilization. As observed in Figure 1, in comparison with WT kAE1, which was predominantly located at the plasma membrane, every condition resulted in the localization of the G701D kAE1 mutant at the cell surface of polarized MDCK cells. Trafficking of the R589H kAE1 mutant was corrected to some extent after low temperature or glycerol incubations, but not by DMSO treatment. To quantitatively assess the extent of the rescue, we counted the percentage of transfected cells with detectable cell surface kAE1 expression in control or treated conditions. We found that although only 14% of G701D kAE1 cells (n = 228) showed cell surface staining in control conditions, this number increased to 54 (n = 179), 65 (n = 142) and 65% (n = 164) after DMSO, glycerol or 30°C incubations for 16 hours, respectively. Surface staining was detectable on 1 (n = 119), 17 (n = 68) or 4% (n = 69) of cells expressing R589H kAE1 after 16 hours of DMSO, glycerol or 30°C incubation, respectively, while only 1% (n = 194) of the cells showed surface staining in control conditions. No improvement in trafficking was seen for the misfolded C479W kAE1 proteins after any treatment. These findings indicate that chemical treatments or low temperature incubations increased the number of cells displaying plasma membrane G701D or R589H kAE1 mutants.

To support these findings, we compared the percentage of kAE1 carrying complex oligosaccharide in lysates from untreated or treated cells on immunoblots. The presence of kAE1 carrying complex oligosaccharide indicates that kAE1 trafficked to the Golgi and beyond. We found that although kAE1 WT showed a constant percentage of complex oligosaccharides upon treatment (71±9% in control conditions, versus 69±4, 68±11 and 68±3% (n = 3, ± SEM) in DMSO, glycerol or 30°C conditions, respectively), the percentage of complex oligosaccharide significantly increased after treatment of kAE1 G701D expressing cells (51±4% in control conditions, versus 69±3, 62±1 and 69±3% (n = 3, ± SEM) in DMSO, glycerol or 30°C conditions, respectively). The percentage of complex oligosaccharide did not significantly increase in treated kAE1 R589H expressing cells (6±1% in control conditions versus 5±1%, 14±7%, 2±1% (n = 3, ± SEM) in DMSO, glycerol or 30°C conditions, respectively). Similarly, no increase was found for kAE1 C479W expressing cells upon treatment (6±1% in control conditions versus 6±1%, 4±1%, 4±1% (n = 3, ± SEM) in DMSO, glycerol or 30°C conditions, respectively). Thus, chemical treatments or low temperature incubation can increase both complex glycosylation and cell surface trafficking of the G701D kAE1 mutant.

### DMSO Chemical Treatment Partially Rescues the Functional Activity of G701D kAE1 Mutant

**Figure 2:**
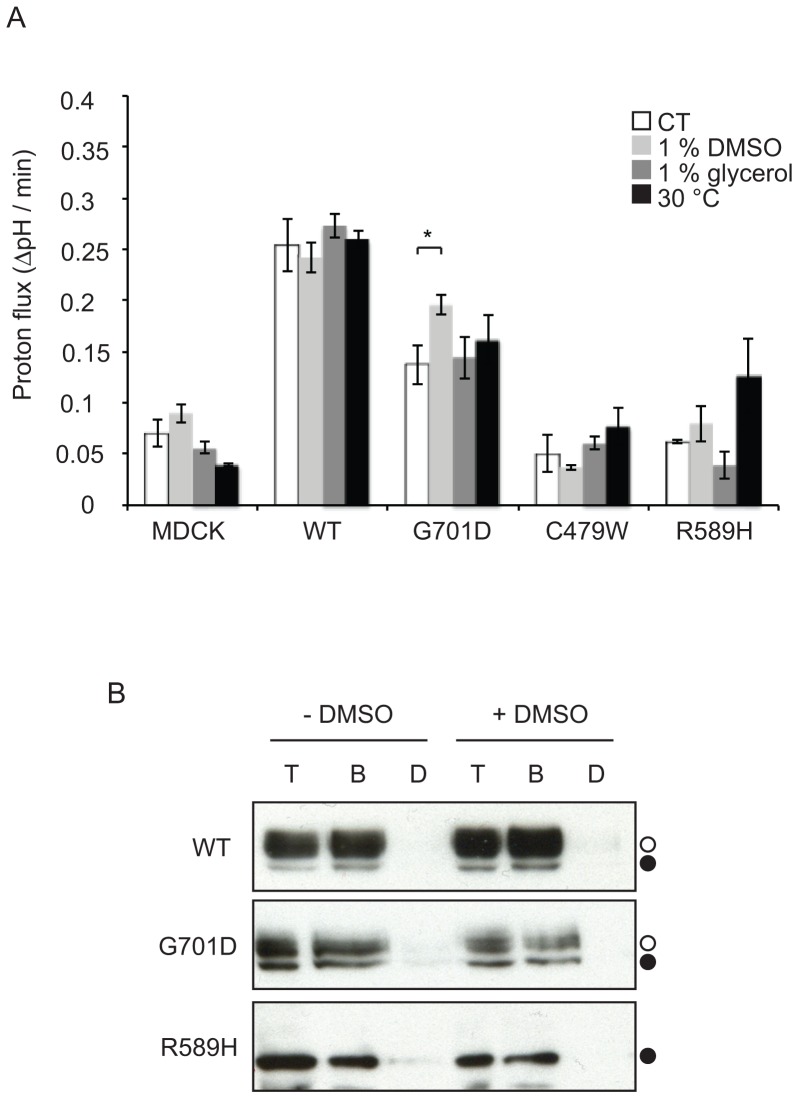
DMSO can partially rescue the function of G701D kAE1. A, MDCK cells expressing WT or kAE1 mutants were grown on glass coverslips and either kept in control conditions or treated for 16 hours with DMSO, glycerol or low temperature. After incubation with 10 µM of the pH sensitive fluorescent probe BCECF-AM, coverslips were placed in a cuvette and perfused with Ringer’s solution containing sodium chloride at room temperature. The buffer was then switched to a chloride-free Ringer’s solution and the increase of BCECF fluorescence recorded. After completion of the experiment, the BCECF fluorescence was calibrated using high potassium buffers containing nigericin at three different pHs. The variation in pH units per min was calculated for each mutant in control or treated conditions. Values are mean ± SEM, n = 3 independent experiments. Asterisk indicates statistically significant difference with control condition (P≤0.05), calculated by one-way ANOVA. B, Cell lysates from MDCK cells expressing kAE1 WT or dRTA mutants and treated with (right panel) and without (left panel) DMSO for 16 hours, were incubated with SITS-Affi-Gel with and without incubation with the free inhibitor H_2_DIDS. After three washes, the proteins were eluted and analyzed by immunoblot using a mouse anti-HA antibody followed by anti-mouse antibody coupled to HRP. T, total cell lysate, B, eluate from SITS-Affi-Gel, D, eluate from SITS-Affi-gel with pre-incubation with free H_2_DIDS. Filled circles correspond to kAE1 proteins carrying complex oligosaccharide, open circles indicate kAE1 proteins carrying high mannose oligosaccharide.

As the chemical treatments with DMSO and glycerol or low temperature incubations all rescued cell surface trafficking of the G701D kAE1 mutant, we next asked whether the rescued mutant was functional at the cell surface. We carried out a functional assay using the ratiometric fluorescence–based pH sensitive dye 2′,7′-Bis(2-carboxyethyl)-5(6)-carboxyfluorescein-acetoxymethyl ester (BCECF-AM). The cells were pre-loaded with BCECF-AM in the presence of chloride, followed by perfusion with a chloride free solution in the presence of extracellular bicarbonate [33]. In these conditions, if kAE1 is functional and at the plasma membrane, entry of bicarbonate through kAE1 leads to an increased intracellular pH and subsequently augments the intracellular BCECF fluorescence. The bicarbonate-chloride transport rate in the initial 30 seconds of activity was measured and compared to non-treated conditions. The various incubations or low temperature incubations did not significantly affect the intrinsic buffer capacity of the cells (see materials and methods). Figure 2 A shows that although all treatments improved G701D kAE1 trafficking, DMSO incubation was the only condition that showed a significant improvement in function at the plasma membrane. Of note, the rescuing effect of the treatments on kAE1 functional activity may be minimized as the functional assays were performed at room temperature and in buffers devoid of chemical chaperones. Interestingly, in control conditions, the G701D dRTA mutant showed a higher anion exchange activity than the negative control cells, consistent with previous studies [25] and with our quantification of cell surface expression of this mutant. Low temperature incubation did not significantly increase the average transport function of the R589H kAE1 mutant.

We next tested whether incubations with DMSO affect the ability of kAE1 dRTA mutants to bind stilbene inhibitors compared to proteins in untreated cells. We compared the ability of kAE1 WT and dRTA mutants to bind SITS-Affi-gel after incubating cells with and without 1% DMSO for 16 hours (Figure 2 B). As previously reported, in MDCK cells, kAE1 is present as two main sub-populations on immunoblots: a high mannose-carrying sub-population present in the ER (low molecular band, closed circles) and a complex glycosylated sub-population that has reached the medial Golgi (high molecular weight band, open circles) [8], [26]. The kAE1 dRTA mutants expressed in cells treated with DMSO displayed binding to the SITS-Affi-gel similar to mutants expressed in untreated cells. These data suggest that DMSO treatment did not grossly alter kAE1 stilbene inhibitor binding site.

Together, our results indicate that rescued trafficking of a kAE1 dRTA mutant by several chemical treatments or low temperature incubation does not necessarily correlate to a detectable restoration of function at the plasma membrane. Thus, not all the treatments are equivalent in fully reverting the detrimental effect of the mutation.

### The Pharmacological Corrector VX-809 also Rescues G701D kAE1 Mutant Trafficking

**Figure 3:**
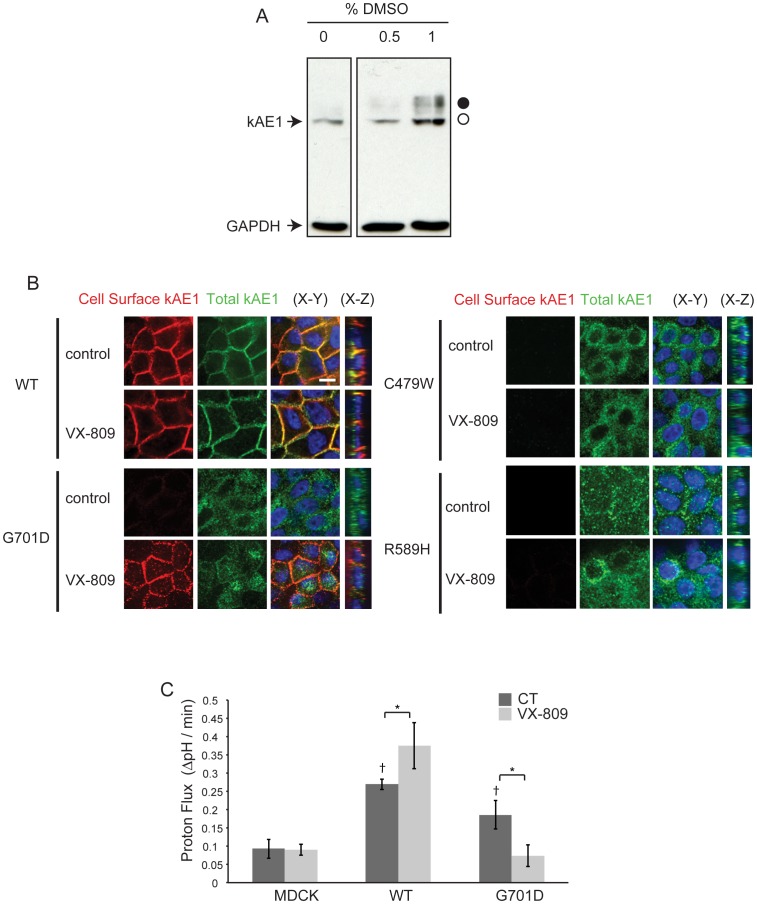
The molecular chaperone VX-809 rescues trafficking but not function of G701D kAE1. A, MDCK cells expressing WT or kAE1 mutant were either kept in control conditions or treated with 0.5 or 1% DMSO for 16 hours prior to lysis. kAE1 proteins present in the lysate were resolved by immunoblot using a mouse anti-HA antibody followed by an anti-mouse antibody coupled to HRP. Glyceraldehyde phosphate dehydrogenase (GAPDH) was detected as a loading control. Filled circles correspond to kAE1 proteins carrying complex oligosaccharide, open circles indicate kAE1 proteins carrying high mannose oligosaccharide. B, MDCK cells expressing WT or kAE1 mutant were either kept in control conditions or treated with 3 µM of the molecular chaperone VX-809 for 24 hours prior to fixation. We then performed a two-step immunostaining as follows: cells were blocked and stained with a mouse anti-HA antibody followed by staining with an Alexa 488 coupled secondary antibody. Cells were then permeabilized and after blocking, stained again with a mouse anti-HA antibody followed by a Cy3 coupled secondary antibody. The samples were examined with a WaveFx spinning disk using a 60×oil immersion objective. The size bar corresponds to 10 µm. C, MDCK cells expressing WT or G701D kAE1 were grown on glass coverslips and either kept in control conditions or treated for 16 hours with 3 µM VX-809. After incubation with 10 µM of the pH sensitive fluorescent probe BCECF-AM, coverslips were placed in a PTI cuvette and perfused with a Ringer’s solution containing sodium chloride. The buffer was then switched to a chloride-free Ringer’s solution and the increase of BCECF fluorescence recorded. After completion of the experiment, the BCECF fluorescence was calibrated using high potassium buffers containing nigericin at three different pHs. The variation of pH units per min was calculated for each mutant in control or treated conditions. Values are mean ± SEM, n = 3 independent experiments. Asterisk indicates statistically significant difference with control condition (P≤0.05), calculated by one-way ANOVA. Dagger signs indicate significant difference (P≤0.05), compared with MDCK in control conditions.

The recently identified pharmacological corrector VX-809 restored trafficking and function of the most common CFTR mutant – deltaF508– to levels where phenotypic improvement was seen in CF patients [22]. We wondered whether this corrector would also have a positive effect on kAE1 mutants’ processing and folding. We determined that 3 µM [prepared in a final 0.4% DMSO, a concentration that did not significantly improve trafficking of kAE1 WT or G701D mutant (Figure 3 A)] is the smallest concentration of VX-809 that provided a noticeable increase in the mature form of G701D kAE1 carrying the complex oligosaccharide compared to the total amount of kAE1 (data not shown). Cell surface immunolabelling experiments indicated that VX-809 rescued trafficking of the G701D kAE1 mutant to the cell surface (Figure 3 B). In contrast, VX-809 treatment had no clear effect on trafficking of the C479W or R589H kAE1 mutants. Thus, VX-809 mimicked the rescuing effect of low temperature incubations or chemical treatment on kAE1 processing to the cell surface. However, functional assays demonstrated that rather than improving the function of the rescued G701D kAE1 at the plasma membrane, VX-809 induced a significant decrease in activity of this mutant (Figure 3 C). Together, Figures 1, 2 and 3 show that trafficking of some dRTA mutants can be partially rescued, however, not all treatments have the ability to restore function of the mutant.

### DMSO Incubation Increases Synthesis of kAE1 Proteins

**Figure 4:**
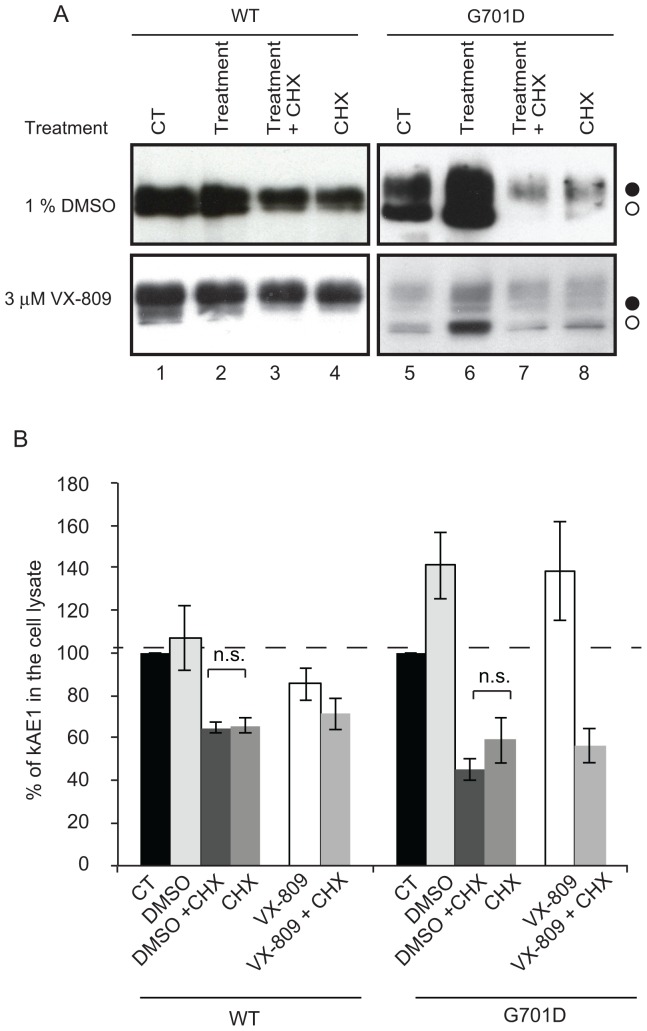
Both DMSO and VX-809 increase synthesis of the G701D kAE1. A, MDCK cells expressing WT or mutant kAE1 were either kept in control conditions or treated for 4 hours with CHX (10 µg/ml), 1% DMSO (top panels) or 3 µM VX-809 (bottom panels) in presence or absence of CHX prior to lysis. After the protein concentration was determined, proteins (9 µg and 6 µg for DMSO and VX-809 treatments, respectively) were resolved by SDS-PAGE then immunobloted and detected using a mouse anti-HA antibody followed by an anti-mouse antibody coupled to HRP. Filled circles correspond to kAE1 proteins carrying complex oligosaccharide, open circles indicate kAE1 proteins carrying high mannose oligosaccharide. B, Histogram showing the percentage of WT or G701D kAE1 proteins present in lysates from cells kept in control conditions or treated as indicated. Values are mean ± SEM, n = 3 independent experiments. N.s. indicates non-statistically significant differences.

We next investigated the mechanism by which DMSO and VX-809 acted on kAE1 trafficking and cell surface processing. We immunoblotted WT or G701D kAE1 mutant from MDCK cells that were either untreated or incubated with DMSO or VX-809 (Figure 4 A, lanes 1–2 & 5–6 and Figure 4 B). When cells were treated for 4 hours with either DMSO or VX-809, comparison of the immunoblot band density revealed that the overall amount of G701D kAE1 was increased by 41±15% (n = 3, ± SEM) and 38±23% (n = 3, ± SEM), respectively, while the amount of WT kAE1 was not significantly changed.

Three possible mechanisms could result in increased kAE1 in treated cells: the treatment either increased protein synthesis, decreased protein degradation or a combination of both. To discriminate between these hypotheses, we treated cells expressing WT or the G701D kAE1 mutant either in control conditions, with CHX to inhibit protein synthesis [36], with CHX and DMSO or with DMSO only for 4 hours (Figure 4). We expected that if DMSO acts by increasing kAE1 synthesis, protein synthesis inhibition using CHX would prevent the rescuing effect of DMSO, and levels of kAE1 protein would be comparable to cells treated with CHX only. Therefore, the intensity of kAE1 bands in “DMSO+CHX” condition (lanes 3 and 7, top panels) should be similar to that of kAE1 in “CHX” conditions (lanes 4 and 8, top panels). As seen in Figure 4 A and B, in lanes 7 and 8, after a 4 hour incubation with DMSO and CHX, G701D kAE1 mutant was detected at a similar amount than in cells treated with CHX only. This indicates that chemical inhibition of protein synthesis completely reverted the effect of DMSO on kAE1 amount. Similar observations were made in cells treated with VX-809 (Figure 4 A, lower panels). The levels of kAE1 remaining in lysates from cells treated with DMSO+CHX were lower than in cells treated with CHX only, possibly indicating that rescued kAE1 proteins are more rapidly degraded than in cells treated with CHX only. However, the difference between “DMSO+CHX” condition versus “CHX” condition, each normalized to control conditions, was not significantly different for either kAE1 WT (66±4% in CHX/control versus 65±3% in DMSO+CHX/control (n = 3, ± SEM)) or G701D (59±11% in CHX/control versus 45±5% in DMSO+CHX/control (n = 3, ± SEM)), indicating that DMSO does not predominantly affect kAE1 proteins degradation. Together, these results support that both DMSO and VX-809 increase the steady-state amount of kAE1 primarily by promoting kAE1 protein synthesis.

## Discussion

In the present work, we determined whether trafficking to the cell surface and function of C479W, R589H or G701D dRTA kAE1 mutants could be rescued after treatment with chemical chaperones (DMSO or glycerol), a pharmacological agent (VX-809) or low temperature incubations. All the treatments partially rescued cell surface trafficking of the G701D kAE1 mutant. Our main finding is that DMSO treated cells expressing G701D kAE1 showed significant increase of functional activity after treatment. Despite limited trafficking rescue of the R589H dRTA mutant to the surface of a few cells after glycerol treatment, there was no significant improvement in its functional activity. The inactive C479W dRTA mutant was not rescued by any treatment tested here. These findings indicate that *(i)* trafficking and function of the Golgi-retained G701D kAE1 dRTA mutant have the potential to be rescued; *(ii)* trafficking of the R589H mutant may be rescued but a folding corrector has yet to be identified, and *(iii)* DMSO, glycerol, VX-809, and low temperature treatments are not equivalent in restoring trafficking and function of dRTA mutants to the cell surface. Our experiments indicate that rather than directly acting on the folding of the mutants, the treatments primarily increased kAE1 expression levels. Thus, we hypothesize that treatments act primarily by overwhelming the quality-control machinery.

The G701D kAE1 mutant is a recessively inherited dRTA mutant that is retained in the Golgi of renal epithelial cells [8]. Patients homozygous for this mutation display only dRTA symptoms independent of RBC features - G701D eAE1 in the RBCs of patients display 98% sulfate influx and 95% of the inhibitor DIDS binding sites compared with WT eAE1 [19]. When expressed in *Xenopus* oocytes, this mutant is inactive unless co-expressed with the erythroid restricted, chaperone-like protein GPA [19]. These previous findings suggested that this mutant is mildly misfolded and that its functional rescue may be possible. In the present study, we show that glycerol, DMSO, VX-809, and low temperature incubations partially rescued G701D kAE1 trafficking to the surface of renal epithelial cells (Figure 1 & 3). However, DMSO is the only treatment that also significantly restored function of the mutant at the cell surface (Figure 2 A). As the functional assays were performed at room temperature and in buffers devoid of chemical chaperones, the rescuing effect of the treatments on kAE1 functional activity may be minimized. Nevertheless, this finding suggests that DMSO incubation may have enabled G701D kAE1 dRTA mutant to achieve a conformation close to WT kAE1 and to the one adopted by G701D kAE1 in the presence of GPA. Interestingly, the G701D kAE1 mutant was rescued by all the treatments we used in this study, suggesting that this Golgi-retained mutant was more “rescuable” than ER retained mutants. As all our constructs contained an external HA epitope, we cannot exclude the possibility that the presence of the epitope affected the way treatments improved trafficking of the mutants. Our experiments indicate that DMSO incubations predominantly increased the overall protein synthesis of the G701D kAE1 mutant (Figure 4), as did incubations with the VX-809 pharmacological chaperone. However, in contrast with VX-809 and other treatments described in this study, DMSO incubations may also promote better folding of this mutant, thereby resulting in the partially rescued function observed at the cell surface. We did not detect an improved ability of the G701D kAE1 mutant to bind the stilbene inhibitor SITS after DMSO treatment (Figure 2 B). However, this assay does not exclude folding improvement at kAE1 transport regions different from the inhibitor binding site. Interestingly, trafficking of this mutant was not rescued by small molecule inhibitors that disrupt the calnexin and Hsc70 chaperone interaction with kAE1 [25]. These findings contrast with the ability of the three treatments tested here to rescue G701D mutant trafficking. Together, they suggest that DMSO, glycerol and VX-809 treatments possibly overwhelm the quality-control machinery by primarily causing over-expression of the G701D mutant, as shown in Figure 4, or, small molecule inhibitors that target chaperones involved in the ER-quality control machinery are ineffective because the G701D kAE1 mutant is largely Golgi localised.

The functionally inactive C479W kAE1 mutant was not rescued by any treatment that we have tested in these experiments, suggesting that the folding of the mutant cannot be improved. The cellular quality-control machinery likely detects this mutant as terminally misfolded, targeting it for degradation regardless of the treatment applied. The C479W mutation is located at the boundary between the third transmembrane domain and the second extracellular loop [27]. Interestingly, in the second extracellular loop, another recessive dRTA mutation, V488M, also impairs proper kAE1 folding and trafficking [21], [37], [38]. The dramatic effect resulting from these two neighboring mutations, C479W and V488M, supports a role for this region in proper folding and suggests that these mutants are likely terminally misfolded.

After glycerol incubations, we observed a negligible increased number of cells with kAE1 R589H mutant at the cell surface by immunofluorescence. This minimal effect aligns with the failure of sodium butyrate treatment and low temperature incubation to significantly rescue trafficking of the same mutant in MDCK I cells [26]. Although this mutant exhibits approximately 50% of function in *Xenopus* oocytes [2], there was no significant functional improvement of this mutant after glycerol incubation, suggesting that glycerol did not rescue trafficking to a sufficient extent to detect functional improvement at the cell surface.

Our experiments indicate that treatments that partially rescued cell surface trafficking (DMSO, glycerol, VX-809 and low temperature incubations) predominantly acted by increasing the steady-state amount of kAE1 within a time as short as 4 hours. This increase might be a general effect of the treatments on increasing cellular protein synthesis. Alternatively, this result may either reflect an improved ability of the cellular chaperones to fold proteins. Glycerol and DMSO incubations protect misfolded membrane proteins from thermal denaturation, aggregation and misfolding [24]. They may allow for tighter packing and stabilization of G701D kAE1 folding intermediates, thus promoting their ability to fold properly and further help their trafficking to the plasma membrane. However, they likely act with subtle differences, as DMSO but not glycerol rescued functional activity of G701D kAE1. Low temperature incubation did partially rescue cell surface trafficking of the G701D kAE1 mutant, but not its activity. As low temperature incubation inhibits proteasome function [39], it suggests that reducing proteasome-mediated degradation would not be enough to treat dRTA patients with the G701D mutation. Patients with the G701D mutation may therefore need a molecular chaperone that would not only improve trafficking but also folding of this mutant.

The VX-809 molecular corrector partially rescued the maturation and chloride transport activity of CFTR deltaF508 in human bronchial epithelial cells [22]. However, although it partially rescued G701D kAE1 dRTA mutant trafficking to the cell surface, there was no improved functional activity observed in our experiments (Figure 3). This indicates that the correcting effect of VX-809 is unique to CFTR deltaF508 and may not apply to other mutant membrane proteins that display trafficking defects. This is consistent with the lack of rescuing effect of this molecular chaperone on trafficking and function of two other mis-trafficking mutants - human ether-a-go-go related K^+^ channel (G601S hERG) and P-glycoprotein (G268V and Y490del P-gp) [22].

Taken together, our experiments demonstrate the potential for trafficking and function of one dRTA mutant, G701D kAE1 to be rescued to the plasma membrane of renal epithelial cells. These findings suggest that extending the investigation to a larger number of molecular chaperones may identify chemicals that could potentially rescue trafficking and function of this mutant. Although prescription of oral alkaline supplements is so far the most cost effective therapeutic treatment for dRTA, our study highlights that prospective alternative treatments for patients suffering from dRTA, as a result of mutated kAE1, can be developed to target the source of the disease, rather than its consequences. Our findings also provide leads for rescuing trafficking of other mutated bicarbonate transporters that cause human diseases.
